# Sleep spindles are reduced in children with Down syndrome and sleep-disordered breathing

**DOI:** 10.1038/s41390-023-02854-1

**Published:** 2023-10-16

**Authors:** Marisha Shetty, Margot J. Davey, Gillian M. Nixon, Lisa M. Walter, Rosemary S. C. Horne

**Affiliations:** 1 Department of Paediatrics, Monash University, Melbourne, VIC Australia; 2 Melbourne Children’s Sleep Centre, Monash Children’s Hospital, Melbourne, VIC Australia

## Abstract

**Background:**

Children with Down syndrome (DS) are at increased risk of sleep-disordered breathing (SDB). We investigated sleep spindle activity, as a marker of sleep quality, and its relationship with daytime functioning in children with DS compared to typically developing (TD) children.

**Methods:**

Children with DS and SDB (*n* = 44) and TD children matched for age, sex and SDB severity underwent overnight polysomnography. Fast or Slow sleep spindles were identified manually during N2/N3 sleep. Spindle activity was characterized as spindle number, density (number of spindles/h) and intensity (density × average duration) on central (C) and frontal (F) electrodes. Parents completed the Child Behavior Check List and OSA-18 questionnaires.

**Results:**

In children with DS, spindle activity was lower compared to TD children for F Slow and F Slow&Fast spindles combined (*p* < 0.001 for all). Furthermore, there were no correlations between spindle activity and CBCL subscales; however, spindle activity for C Fast and C Slow&Fast was negatively correlated with OSA-18 emotional symptoms and caregiver concerns and C Fast activity was also negatively correlated with daytime function and total problems.

**Conclusions:**

Reduced spindle activity in children with DS may underpin the increased sleep disruption and negative effects of SDB on quality of life and behavior.

**Impact:**

- Children with Down syndrome (DS) are at increased risk of sleep-disordered breathing (SDB), which is associated with sleep disruption affecting daytime functioning.
- Sleep spindles are a sensitive marker of sleep quality.
- We identified for the first time that children with DS had reduced sleep spindle activity compared to typically developing children matched for SDB severity.
- The reduced spindle activity likely underpins the more disrupted sleep and may be associated with reduced daytime functioning and quality of life and may also be an early biomarker for an increased risk of developing dementia later in life in children with DS.

## Introduction

Obstructive sleep-disordered breathing (SDB) describes a spectrum of respiratory disorders ranging from primary snoring (PS), which is not associated with significant desaturation or sleep fragmentation, to obstructive sleep apnea (OSA), which is characterized by repetitive hypoxia, hypercarbia and/or sleep disruption.^1^ SDB is very common in typically developing (TD) children, with the prevalence of habitual snoring (snoring often or always) being reported in 1.5–27.6% and OSA in 1–6% of children.^1^ Studies have identified that SDB of all severities, including PS, are associated with adverse effects on daytime behavior and functioning, including poorer school performance.^2^ It is hypothesized that these adverse outcomes are mediated by the repetitive hypoxia and sleep disruption that are associated with SDB.^3^ However, studies using both conventional polysomnographic (PSG) measurements of sleep macro-architecture, i.e., a measure of the structure of sleep, including total sleep time and the percentage of total sleep spent in the two sleep states nonrapid eye movement (NREM) and rapid eye movement (REM) sleep, as well as sleep microarchitecture, i.e., the spectral analysis of the electroencephalogram (EEG) which assesses more subtle changes in sleep, have not identified major changes in children with SDB compared to non-snoring control children.^4,5^

To investigate the mechanisms that may underpin the adverse consequences of SDB on behavior and neurocognition, researchers have begun to investigate specific elements of the sleep EEG that may be markers of sleep disruption not identified using routine clinical studies. A potential candidate is the sleep spindle. Sleep spindles are a hallmark waveform of N2 sleep and represent an oscillating electrical potential in the brain. They have a characteristic frequency in the sigma range of 11–16 Hz, but are usually 12–14 Hz and last from 1 to 3 s in duration.^6^ On the EEG, spindles are visually identified as sinusoidal waves that have a “crescendo-decrescendo” pattern.^7^ Sleep spindles can be divided into two distinct types based on their frequency: slow spindles (9–<13 Hz) that occur maximally over frontal regions and fast spindles (>13–16 Hz), which dominate in central and parietal regions.^8^ Spindles are generated in the thalamus and synchronized in the cortex and, therefore, reflect thalamocortical activity.^9^ Spindles play a primary role in protecting the sleeping brain from external sensory stimuli and can serve as markers of sleep integrity.^10,11^ Spindles are thought to play an important role in the consolidation and re-organization of memories by preventing sleep fragmentation.^12,13^ Studies in healthy children have shown that sleep spindle activity is associated with different aspects of cognitive performance, although the direction of these relationships differs between studies.^14–16^ Reduced spindle activity has also been demonstrated in adults with Alzheimer’s disease.^17^

The incidence of OSA is far higher in children with Down syndrome (DS), where the condition has been reported in 31–97%, depending on patient selection criteria, definitions and methodologies used.^18^ The distinct dysmorphic features of DS, such as mid-face and mandibular hypoplasia, relatively large and medially positioned tonsils, and relative macroglossia result in a significant reduction in the size of the upper airway in children with DS when compared to TD children, thus increasing the risk of SDB.^19,20^ In addition, obesity and hypotonia are common in DS, and potentially contribute to the collapse of the upper airway during sleep and the risk of SDB.^20^ Both parent-reported symptoms^21,22^ and PSG studies^23–27^ have linked SDB with reduced daytime executive and language functioning and cognition in children with DS. Previously, we have shown that sleep in children with DS is more disrupted with more wake after sleep onset compared to TD children matched for SDB severity.^28^ In this study, we also identified that the children with DS had lower sigma power in N2 and N3 sleep compared to TD children, suggesting that sleep spindles may be affected.^28^ Thus, the aim of this study was to specifically investigate sleep spindle activity and its relationship with sleep quality and daytime functioning in children with DS compared to TD children matched for SDB severity.

## Methods

Ethical approval for this study was provided by the Monash University and Monash Health Human Research Ethics Committees (12276B, 14024B, 15048A). Written informed consent was obtained from parents and verbal assent from children aged over 7 years. No monetary incentive was provided for participation.

### Subjects

Children with DS aged 3–19 years referred for assessment of SDB were recruited between May 2016 and March 2018. Control TD children with SDB had been referred clinically for assessment of SDB and were identified from our research database of TD children studied between June 2013 and December 2016. Each child with DS was matched to a TD child by age and sex, and with the same SDB severity based on the obstructive apnea-hypopnea index (OAHI): PS (OAHI ≤1 event/h), mild OSA (OAHI of >1–≤5 events/h), moderate OSA (OAHI of >5–≤ 10 events/h) or severe OSA (>10 events/h). The moderate and severe (MS) groups were combined. For details on matching, please see the Supplementary information.

### Protocol

All children underwent overnight attended PSG using standard pediatric recording techniques.^29^ Prior to the PSG study, height and weight were measured and body mass index (BMI) *z*-score was calculated.^30^ Neck, waist and hip circumference were also measured. Obesity was defined as ≥95th percentile (BMI *z*-scores ≥1.65) and overweight as ≥85th percentile (BMI *z*-scores ≥1.04). For details on matching, please see the Supplementary information.

Electrophysiological signals were recorded using a commercially available PSG system (E-Series or Grael, Compumedics, Melbourne, Australia). See the Supplementary information for full details.

### Sleep spindles analysis

Sleep spindles were identified manually using Compumedics Profusion 3.0 software (Melbourne, Australia) from the C4-A1 and F4-A1 EEG channels during N2 and N3 sleep.^31^

Spindles were then categorized and labeled as either being F4 or C4 Slow or F4 or C4 Fast spindles, and F4 or C4 Slow&Fast spindles combined. Spindle density was calculated as the number of spindles per minute of N2 or N3 sleep; spindle intensity was calculated as the product of spindle density and average spindle duration. For more details on spindles analysis, see the Supplementary information.

### Questionnaires

Parents of both the children with DS and the TD children also completed the OSA-18^32^ and the Child Behavior Check List (CBCL).^33^ In addition, the parents of the children with DS also completed the Adaptive Behavior Assessment System, Second Edition (ABAS-II),^34^ the Pediatric Sleep Problem Survey Instrument (PSSI)^35^ and the Epworth Sleepiness Scale for Children and Adolescents (ESS-CHAD).^36^ See the Supplementary information for full details of the questionnaires used.

### Statistical analysis

All statistical analyses were performed with Sigma Plot (Systat Software Inc Version 14.5). Data were first tested for normality and equal variance. Differences between DS and TD groups as a whole, and within each severity group were compared with Student’s *t*-tests or Mann–Whitney rank sum tests and differences between SDB severity groups within each group (DS or TD) for demographics, sleep and respiratory parameters, OSA-18 and CBCL questionnaires and spindle properties were compared using a one-way analysis of variance (ANOVA) with Bonferroni post hoc tests if normally distributed or a Kruskal–Wallis one-way ANOVA on the ranks with Dunn’s post hoc tests if not normally distributed. Associations between spindle activity indices, and OAHI and questionnaire scores were tested using Spearman rank-order correlations. All *p* values are two-tailed, with significance taken at *p* < 0.05. Parametric data are presented as mean ± standard deviation (SD) and non-parametric data as median and interquartile range (IQR).

## Results

### Demographic, sleep and respiratory characteristics

Demographic data are presented in Table 1. SDB severity groups were matched for sex with the exception of the Mild OSA group as mentioned in the Supplementary information. There were no differences between the TD and DS groups for any of the other demographic variables measured. There were also no statistical differences between groups for the number of children who were obese (DS: 33%; TD: 23%) or overweight (DS: 10%; TD: 16%).

**Table 1:** Demographics of Down syndrome and control children.

	All children		Primary snoring		Mild OSA		Moderate/severe OSA	
	TD	DS	TD	DS	TD	DS	TD	DS
*N*	44	44	11	11	13	13	20	20
Sex	23 F/21 M	25 F/19 M	6 F/5 M	6 F/5 M	5 F/8 M	7 F/6 M	12 F/8 M	12 F/8 M
Age (years)	7.6 (5.2–11.1)	8.4 (5.3–12.8)	5.8 (4.6–12.1)	5.5 (4.0–12.2)	6.3 (5.00–9.8)	6.7 (5.4–9.3)	8.5 (5.8–12.0)	10.5 (6.3–15.7)
BMI *Z*-score	0.9 (0.1–1.4)	0.9 (0.4–1.8)	0.9 (0.4–1.2)	0.8 (0.5–1.3)	0.7 (0.1–1.2)	0.7 (0.2–1.9)	1.2 (0.1–2.4)	1.3 (0.6–2.3)
Neck circumference (cm)	28.0 (26.6–33.0)	29.8 (27.1–36.0)	27.0 (26.0–30.0)	28.5 (27.0–34.3)	28.0 (26.5–33.5)	28.0 (27.1–31.0)	30.5 (27.0–36.8)	32.0 (28.1–44.0)
Waist circumference (cm)	61.5 (57.0–77.8)	61.0 (54.9–78.5)	61.0 (58.0–64.0)	59.0 (53.0–63.0)	59.0 (57.0–72.0)	56.50 (53.3–64.0)	72.5 (56.0–98.0)	69.5 (57.0–100.0)
Hip circumference (cm)	65.0 (59.3–88.3)	68.0 (58.0–87.8)	64.0 (60.0–74.0)	63.0 (57.0–77.0)	63.0 (59.0–77.5)	63.0 (57.3–71.8)	79.0 (62.3–107.5)	81.0 (64.0–103.0)

Values are median and IQR.

Sleep characteristics are presented in Table 2. Time in bed was longer in the children with DS as a whole and in each SDB severity group compared with TD children. Sleep period time was longer in the children with DS as a whole (*p* < 0.01) and in the MS OSA group (*p* < 0.05); however, there were no differences in total sleep time (TST) between groups. Percent TST spent in N2 sleep and REM latency were also longer in the children with DS as a whole and in the MS OSA group. WASO was also greater in the children with DS compared to the TD children as a whole.

**Table 2:** Sleep characteristics in children with Down syndrome and typically developing children.

	All children		Primary snoring		Mild OSA		Moderate/severe OSA	
	TD	DS	TD	DS	TD	DS	TD	DS
Time in bed (min)	495.8 (476.3–516.9)	527.8*** (516.0–548.8)	490.5 (465.5–522.0)	528.0*** (500.5–571.0)	513.5 (496.5–520.8)	535.0* (525.5–566.0)	488.0 (465.9–516.9)	524.3** (508.5–531.9)
Sleep period time (min)	470.3 (442.4–492.3)	494.3** (465.6–519.9)	452.0 (435.5–493.5)	488.5 (471.0–527.0)	483.5 (468.5–498.25)	516.5 (441.0–528.8)	454.8 (435.0–482.5)	488.3* (465.6–513.3)
Total sleep time (min)	428.0 (394.1–467.3)	430.3 (380.5–469.9)	428.5 (389.0–455.0)	432.5 (369.5–480.5)	459.5 (417.3–478.8)	430.5 (410.0–466.3)	406.8 (359.1–466.0)	423.8 (338.8–477.9)
Sleep latency (min)	22.5 (14.4–43.9)	20.8 (11.5–47.625)	24.0 (18–42)	37.5 (4–52.5)	22.0 (10.8–43.0)	16.0 (7.8–56.5)	19.3 (12.9–52.375)	21.3 (14.1–46.6)
REM latency (min)	120.3 (92.8–160.9)	162.5** (128.5–222.0)	105.0 (83.5–123)	148.0 (82.5–197.5)	114.0 (91.25–140.5)	177.5* (135.3–211.5)	149.3 (116.3–219.3)	162.5 (134.5–237.5)
Sleep efficiency (%)	86.0 (79.4–91.4)	81.9 (73.9–90.6)	87.3 (83.6–90.2)	83.0 (70.2–91.9)	88.6 (84.5–93.7)	82.8 (76.05–87.4)	84.8 (75.8–91.1)	79.6 (69.8–91.7)
Wake after sleep onset (%)	6.0 (4–13.8)	10.5** (5.3–19.5)	8.0 (4.0–10.0)	9.0 (4.0–22.0)	4.0 (2.5–13)	10.0 (7.5–17.5)	8.5 (4.0–17.0)	12.0 (5.0–21.8)
N1 %TST	6.9 (4.6–9.6)	6.1 (2.7–9.4)	5.9 (2.3–6.9)	7.6 (3.8–13.2)	5.2 (3.7–9.0)	3.8 (1.4–7.5)	7.2 (5.9–12.8)	7.2 (3.3–10.8)
N2 %TST	44.8 (40.4–47.5)	50.5** (44.3–55.9)	46.6 (38.5–56.9)	50.4 (46.0–57.8)	46.7 (45.2–48.2)	50.6 (43.9–56.6)	43.1 (39.8–45.1)	50.9** (43.9–55.7)
N3 %TST	28.6 (24.3–31.4)	25.4 (19.9–33.7)	25.0 (19.9–36.4)	25.3 (20.6–31.5)	26.0 (22.5–29.4)	26.1 (20.5–34.6)	29.4 (27.1–33.7)	24.9 (18.9–33.5)
NREM sleep (%)	80.9 (79.4–83.0)	82.0 (77.7–88.5)	80.8 (77.9–83.0)	85.1 (80.6–91.1)	80.6 (79.3–82.0)	81.2 (78.1–86.1)	81.9 (79.5–85.2)	82.6 (76.8–88.8)
REM sleep (%)	19.2 (17.0–20.6)	18.0 (11.5–22.3)	19.2 (17.0–22.1)	14.9 (8.9–19.4)	19.4 (18.0–20.7)	18.8 (14.0–21.9)	18.1 (14.8–20.6)	17.5 (11.2–23.2)
PLM (TST) (events/h)	0.0 (0.0–1.3)	0.0 (0.0–0.5)	0.0 (0.0–1.3)	0.5 (0.0–1.5)	0.6 (0.0–1.4)	0.0 (0.0–0.7)	0.0 (0.0–1.2)	0.0 (0.0–0.0)

Values are median and IQR.

*p < 0.05, **p < 0.01, ***p < 0.001 DS vs TD.

Respiratory characteristics are presented in Table 3. Although children were matched for SDB severity, REM RDI was greater in the children with DS in the group as a whole and also in the Mild and MS OSA groups compared to the TD children. Measures of oxygen desaturation were all greater in the children with DS compared to the TD children. Due to the definition of the SDB severity groups, OAHI, RDI, ArI, REM RDI and measures of desaturation were different between the SDB severity groups.

**Table 3:** Respiratory characteristics in children with Down syndrome and typically developing children.

	All children		Primary snoring		Mild OSA		Moderate/severe OSA	
	TD	DS	TD	DS	TD	DS	TD	DS
*N*	44	44	11	11	13	13	20	20
OAHI (events/h)	4.2 (0.6–11.8)	4.2 (1.0–12.7)	0.3 (0.0–0.3)	0.1 (0.0–0.4)	2.3 (1.3–3.7)	2.3 (1.4–3.5)	13.1^†††###^ (7.8–28.6)	13.5^†††###^ (6.6–29.0)
CRDI (events/h)	1 (0.6–2.2)	1.6 (0.9–3.2)	0.7 (0.4–1)	1.6 (0.5–2.3)	1.4 (0.9–2.3)	1.9 (1.4–3.5)	1.2 (0.625–2.7)	1.4 (0.7–4.5)
RDI (events/h)	5.9 (2.0–14.2)	8.0 (3.2–16.0)	0.9 (0.4–1.3)	2.6* (1.8–3.2)	4.2 (2.6–5.7)	4.6 (3.4–8.0)	14.6^†††##^ (9.8–31.9)	18.0^†††###^ (12.3–30.0)
Arousal index (events/h)	12.8 (10.5–19.9)	13.9 (11.9–18.0)	9.9 (9.0–11.1)	11.2 (9.5–13.7)	12.6 (9.4–13.3)	12.4 (9.7–14.5)	19.7^†††#^ (12.3–22.9)	17.2^††##^ (14.0–27.2)
REM RDI (events/h)	4.9 (2.3–13.3)	17.0*** (5.9–28.6)	2.3 (0.7–3.8)	5.1 (2.0–7.8)	4.0 (1.5–6.3)	15.0*** (8.3–19.7)	14.2^†††###^ (6.3–39.5)	29.0*^†††##^ (20.4–50.0)
SpO_2_ Nadir (%)	91.0 (87.3–94.0)	88.0** (86.0–90.0)	94.0 (90.0–96.0)	89.0 (88.0–91.0)	92.0 (88.5–94.0)	89.0 (86.5–90.0)	89.5^††^ (83.3–92.0)	86.0^†^ (80.8–88.0)
Average SpO_2_ drop (%)	3.0 (3.0–4.0)	4.0* (3.0–5.0)	3.0 (3.0–3.0)	4.0 (3.0–5.0)	3.0 (3.0–4.0)	4.0 (3.0–4.0)	3.0 (3.0–4.8)	4.0 (3.0–5.0)
SpO_2_ <90% (events/h)	0.0 (0.0–0.1)	0.1** (0.0–0.6)	0.0 (0.0–0.0)	0.1* (0.0–0.3)	0.0 (0.0–0.1)	0.0 (0.0–0.2)	0.05 (0.0–0.7)	0.50*^††^ (0.1–2.0)
SpO_2_ ≥4% drop (events/h)	1.0 (0.3–3.4)	2.4** (1.4–6.2)	0.3 (0.0–0.4)	1.4*** (0.6–1.8)	0.7 (0.4–1.6)	2.1* (1.0–3.3)	3.8^†††##^ (1.3–11.4)	6.8^†††##^ (3.5–10.3)
Average TcCO_2_ TST (mmHg)	43.1 (41.0–47.9)	45.7 (41.1–48.5)	45.5 (41.9–48.4)	43.6 (41.0–46.0)	42.2 (38.6–49.1)	46.3 (40.4–50.1)	43.2 (39.7–47.4)	48.0 (41.8–49.9)

Values are median and IQR.

*p < 0.05, **p < 0.01, ***p < 0.001 DS vs TD.

^†^*p* < 0.05, ^††^*p* < 0.01, ^†††^*p* < 0.001 vs PS.

^#^*p* < 0.05, ^##^*p* < 0.01, ^###^*p* < 0.001 vs mild OSA.

### Differences in spindle indices between children with DS and TD children

**Fig. 1:**
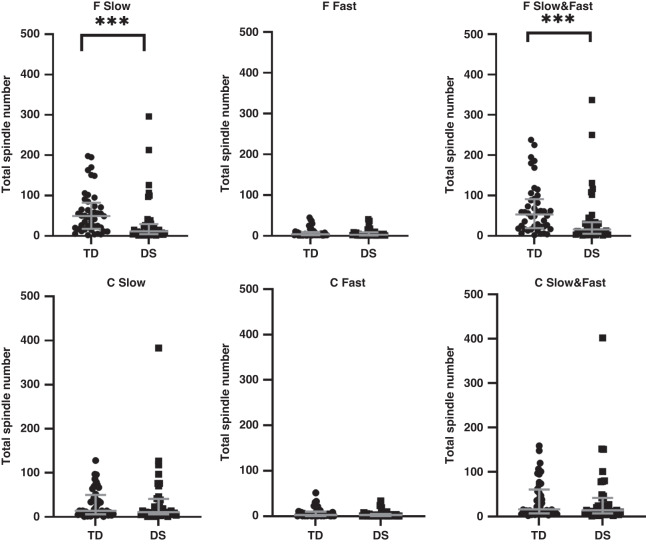
Sleep spindle number. Comparison of sleep spindle number in frontal (F) and centroparietal (C) regions in typically developing (TD) children and children with Down syndrome (DS).

**Fig. 2:**
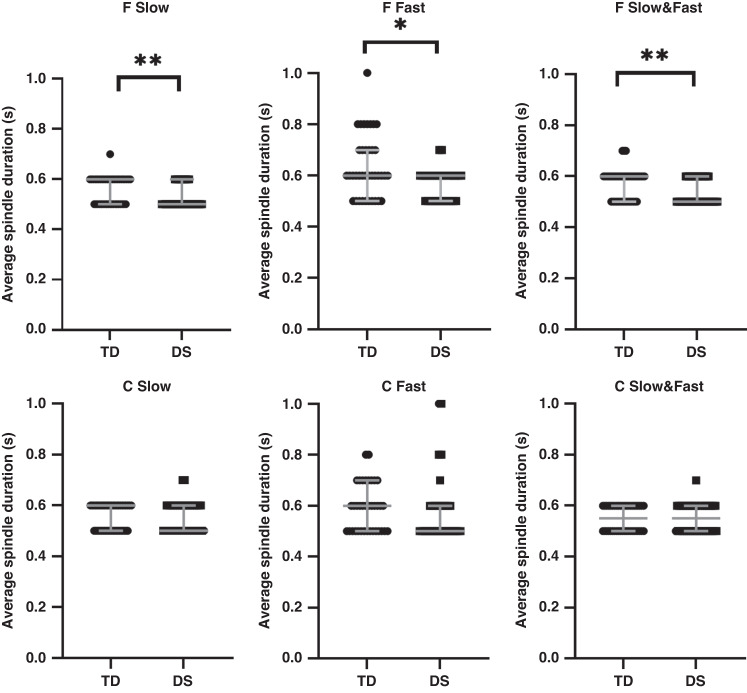
Sleep spindle duration. Comparison of sleep spindle duration in frontal (F) and centroparietal (C) regions typically developing (TD) children and children with Down syndrome (DS).

**Fig. 3:**
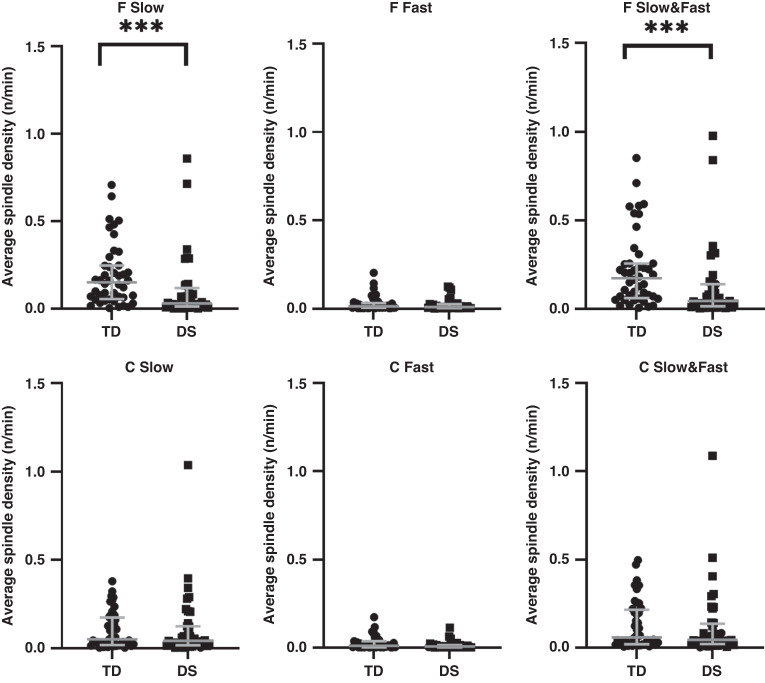
Sleep spindle density. Comparison of sleep spindle density in frontal (F) and centroparietal (C) regions typically developing (TD) children and children with Down syndrome (DS).

**Fig. 4:**
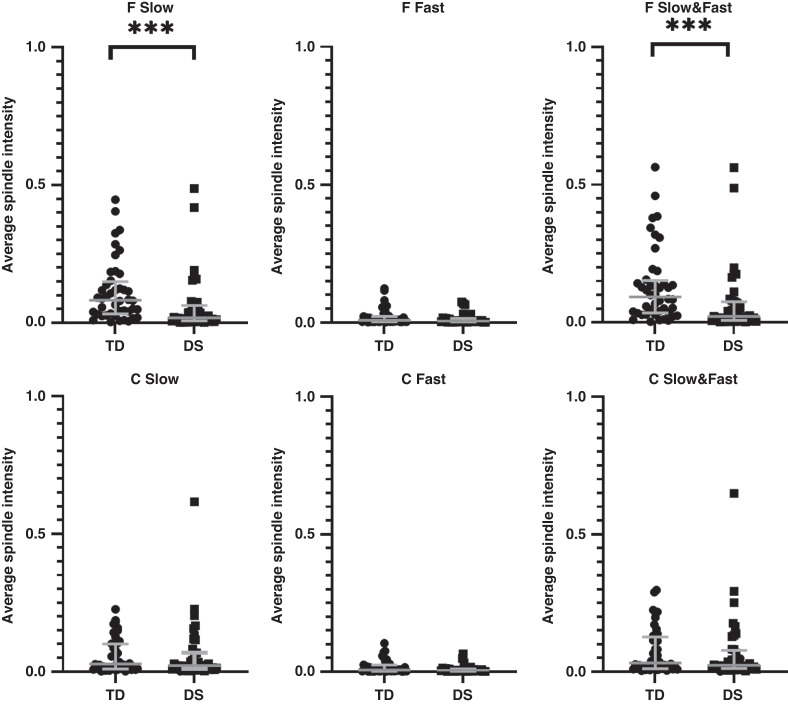
Sleep spindle intensity. Comparison of sleep spindle intensity in frontal (F) and centroparietal (C) regions typically developing (TD) children and children with Down syndrome (DS).

In the group as a whole, spindle duration was shorter in the children with DS for both Slow (*p* < 0.01) and Fast (*p* < 0.05) and combined Slow and Fast (*p* < 0.01) spindles measured from frontal electrodes; however, there were no differences in spindle duration measured from central electrodes (Fig. 1). Spindle numbers (Fig. 2), density (Fig. 3) and intensity (Fig. 4) were all significantly lower in the children with DS compared to the TD children for F Slow and F Slow&Fast spindles combined (*p* < 0.001 for all). There were no differences between groups for spindle indices recorded from central areas. We noted that more children with DS had no spindles identified, with 9% compared to 0% of TD children having no F Slow and 11% compared to 0% having no C Slow spindles. For Fast spindles, 27% of children with DS had none recorded on frontal electrodes compared to 14% of TD children and 30% compared to 16% on centroparietal electrodes; however, these differences did not reach statistical significance. Two children with DS had no spindles recorded in either location.

Differences in spindle duration, total and median number of spindles, spindle density and spindle intensity are compared between SDB severity groups for the F4/C4 Slow and F4/C4 Fast and F4/C4 Fast and Slow combined for the TD children and children with DS in Table 4. Spindle duration was shorter in the children with DS compared to TD children in the PS group for both F Fast and F Slow&Fast (*p* < 0.05 for both) and in the Mild OSA group for both F Slow and F Slow&Fast in the Mild OSA group (*p* < 0.05 for both). In the Mild OSA group, children with DS had fewer F Slow (*p* < 0.001), F Slow&Fast (*p* < 0.01) and C Fast (*p* < 0.05) spindles and spindle density and intensity were also significantly lower compared to TD children. In addition, F Fast spindle density was also lower in the DS children with Mild OSA (*p* < 0.01).

**Table 4:** Comparison of spindle duration, number, density and intensity in children with Down syndrome and typically developing children.

Spindle type			F Slow	F Fast	F Slow and F Fast	C Slow	C Fast	C Slow and C Fast
*Spindle duration*								
PS (*N* = 11)	TD	Median (Q1–Q3)	0.6 (0.5–0.6)	0.6 (0.5–0.8)	0.6 (0.5–0.6)	0.6 (0.5–0.6)	0.6 (0.5–0.7)	0.6 (0.5–0.6)
	DS	Median (Q1–Q3)	0.5 (0.5–0.6)	0.5* (0.5–0.6)	0.5* (0.5–0.6)	0.5 (0.5–0.6)	0.5 (0.5–0.6)	0.5 (0.5–0.6)
Mild OSA (*N* = 13)	TD	Median (Q1–Q3)	0.6 (0.5–0.6)	0.6 (0.5–0.7)	0.6 (0.5–0.6)	0.5 (0.5–0.6)	0.5 (0.5–0.7)	0.5 (0.5–0.6)
	DS	Median (Q1–Q3)	0.5*^†^ (0.5–0.5)	0.6 (0.5–0.6)	0.5* (0.5–0.5)	0.6 (0.5–0.6)	0.5 (0.5–0.6)	0.6 (0.5–0.6)
MS OSA (*N* = 20)	TD	Median (Q1–Q3)	0.6^††^ (0.5–0.6)	0.6 (0.6–0.8)	0.6 (0.5–0.6)	0.6 (0.5–0.6)	0.6 (0.5–0.6)	0.6 (0.5–0.6)
	DS	Median (Q1–Q3)	0.5^†‡^ (0.5–0.6)	0.6 (0.5–0.6)	0.6 (0.5–0.6)	0.5 (0.5–0.6)	0.5 (0.5–0.7)	0.6 (0.5–0.6)
*Spindle number*								
PS (*N* = 11)	TD	Number	716	97	813	352	91	443
		Median (Q1–Q3)	64^††^ (35–91)	2 (1–9)	68 (36–115)	12^†^ (2–40)	3 (1–22)	13 (5–57)
	DS	Number	405	73	478	641	75	716
		Median (Q1–Q3)	8 (4–23)	2 (1–9)	11 (5–28)	7^††^ (5–76)	3 (1–5)	9 (6–80)
Mild OSA (*N* = 13)	TD	Number	801	81	882	313	54	367
		Median (Q1–Q3)	36^†††‡^ (21–94)	2 (2–9)	38 (21–97)	12^†^ (4–45)	1 (1–6)	12 (5–50)
	DS	Number	220	33	253	165	11	176
		Median (Q1–Q3)	7*** (1–20)	1 (0–4)	7** (2–27)	7 (1–24)	0* (0–1)	8 (2–25)
MS OSA (*N* = 20)	TD	Number	1092	157	1249	713	172	885
		Median (Q1–Q3)	46^†††^ (15–58)	5 (1–8)	49 (17–62)	25^†††^ (9–69)	5 (1–9)	29 (11–74)
	DS	Number	758	123	881	585	74	659
		Median (Q1–Q3)	17*^†^ (4–32)	2 (0–7)	23 (5–42)	13 (7–44)	3 (1–4)	15 (10–45)
*Spindle density*								
PS (*N* = 11)	TD	Median (Q1–Q3)	0.16^††^ (0.09–0.33)	0.01 (0.00–0.04)	0.20 (0.13–0.46)	0.03^†^ (0.01–0.16)	0.01 (0.00–0.07)	0.04 (0.02–0.26)
	DS	Median (Q1–Q3)	0.02 (0.01–0.07)	0.01 (0.01–0.03)	0.03 (0.01–0.09)	0.02^††^ (0.01–0.29)	0.01 (0.0–0.02)	0.03 (0.02–0.30)
Mild OSA (*N* = 13)	TD	Median (Q1–Q3)	0.10^††^ (0.06–0.27)	0.01 (0.00–0.03)	0.11 (0.06–0.28)	0.03 (0.01–0.14)	0.00 (0.00–0.02)	0.03 (0.02–0.15)
	DS	Median (Q1–Q3)	0.02*** (0.01–0.06)	0.01* (0.00–0.01)	0.02** (0.01–0.08)	0.02 (0.00–0.06)	0.00* (0.00–0.00)	0.03 (0.00–0.07)
MS OSA (*N* = 20)	TD	Median (Q1–Q3)	0.15^†††^ (0.05–0.23)	0.02 (0.00–0.03)	0.16 (0.05–0.25)	0.08^††^ (0.03–0.22)	0.01 (0.00–0.03)	0.10 (0.04–0.24)
	DS	Median (Q1–Q3)	0.06^†^ (0.01–0.14)	0.01 (0.00–0.02)	0.07 (0.01–0.15)	0.04^†^ (0.02–0.21)	0.01 (0.00–0.02)	0.05 (0.03–0.20)
*Spindle intensity*								
PS (*N* = 11)	TD	Median (Q1–Q3)	0.08^††^ (0.05–0.18)	0.00 (0.00–0.02)	0.13 (0.07–0.27)	0.02^†^ (0.01–0.10)	0.00 (0.00–0.04)	0.02 (0.01–0.14)
	DS	Median (Q1–Q3)	0.01 (0.01–0.04)	0.00 (0.00–0.01)	0.02 (0.01–0.04)	0.01^††^ (0.01–0.17)	0.00 (0.00–0.01)	0.02 (0.01–0.18)
Mild OSA (*N* = 13)	TD	Median (Q1–Q3)	0.06^††^ (0.03–0.16)	0.00 (0.00–0.02)	0.06 (0.03–0.17)	0.02 (0.01–0.08)	0.00 (0.00–0.02)	0.02 (0.01–0.09)
	DS	Median (Q1–Q3)	0.01** (0.00–0.03)	0.00 (0.00–0.01)	0.01** (0.00–0.04)	0.01 (0.00–0.03)	0.00* (0.00–0.01)	0.01 (0.00–0.04)
MS OSA (*N* = 20)	TD	Median (Q1–Q3)	0.08^†††^ (0.03–0.12)	0.01 (0.00–0.02)	0.09 (0.03–0.13)	0.05^†††^ (0.02–0.13)	0.01 (0.00–0.02)	0.05 (0.02–0.15)
	DS	Median (Q1–Q3)	0.03^†^ (0.01–0.07)	0.00 (0.00–0.01)	0.04 (0.01–0.08)	0.02 (0.01–0.10)	0.00 (0.00–0.01)	0.03 (0.02–0.11)

Values are median and IQR.

*p < 0.05, **p < 0.01, ***p < 0.001 TD compared to DS.

^†^*p* < 0.05, ^††^*p* < 0.01, ^†††^*p* < 0.001 fast vs slow spindles.

^‡^*p* < 0.05 frontal vs centroparietal spindles.

In the TD children, in all three SDB severity groups, there were significantly more Slow than Fast spindles identified, with increased spindle density and intensity recorded from frontal and central electrodes (Table 4). In contrast, in the children with DS, there were only more Slow than Fast spindles, with significantly greater density and intensity, recorded from frontal electrodes in the MS OSA group. More Slow than Fast spindles, with increased spindle density and intensity, were also recorded from centroparietal regions in both groups of children. The only difference in spindle duration identified was in the children with DS and Mild OSA, where Fast spindles were longer than Slow spindles in the centroparietal region (*p* < 0.05).

The only regional difference in spindle characteristics observed was significantly more Slow spindles in the frontal compared to centroparietal region in the TD children with Mild OSA (*p* < 0.05).

### Correlations between spindle activity and sleep-disordered breathing severity

There were no significant correlations between OAHI or Arousal Index and spindle number, density or intensity for either Fast or Slow spindles in either C4 or F4 in either the TD children or the children with DS; however, duration of F Fast spindles was positively correlated with Arousal Index in the TD children (*r* = 0.33, *p* < 0.05).

### Correlations between spindle activity and behavior and quality of life

Results of the CBCL and OSA-18 questionnaires of the children with TD and DS children are presented in Table 5. There were no differences between scores for either the CBCL or OSA-18 for TD children compared to children with DS either as a whole group or with each SDB severity group. There was no difference between SDB severity groups for either the TD children or children with DS on the CBCL. As expected, OSA-18 scores were higher in the children with more severe SDB with sleep disturbance and total scores being higher in the MS OSA group compared to the PS group, and caregiver concern and total scores being higher in the Mild OSA group compared to the PS group (*p* < 0.05 for all), in the children with DS. In the TD children sleep disturbance scores were higher in the Mild OSA group compared to the PS group (*p* < 0.05).

**Table 5:** Comparison of CBCL and OSA-18 questionnaires between typically developing children (TD) and children with Down syndrome (DS) and results of the ABAS-II, PSSI and ESS-CHAD questionnaires in the children with DS.

	All children		Primary snoring		Mild OSA		Moderate/severe OSA	
*Child Behavior Check List*								
	TD (*N* = 39)	DS (*N* = 39)	TD (*N* = 11)	DS (*N* = 11)	TD (*N* = 13)	DS (*N* = 10)	TD (*N* = 15)	DS (*N* = 18)
Internalizing	54.3 ± 11.0	55.0 ± 10.8	53.8 ± 8.9	53.2 ± 11.8	54.2 ± 12.0	54.1 ± 12.6	54.7 ± 12.1	56.6 ± 9.5
Externalizing	51.1 ± 11.8	55.0 ± 9.8	50.5 ± 11.4	54.7 ± 7.9	49.7 ± 12.0	58.1 ± 13.3	52.6 ± 12.4	53.4 ± 8.7
Total problems	50.7 ± 11.8	55.5 ± 10.0	50.3 ± 11.3	53.2 ± 11.1	50.2 ± 12.4	57.4 ± 12.3	51.6 ± 12.4	55.9 ± 8.2
*OSA-18*								
	TD (*N* = 40)	DS (*N* = 44)	TD (*N* = 11)	DS (*N* = 11)	TD (*N* = 13)	DS (*N* = 10)	TD (*N* = 16)	DS (*N* = 20)
Sleep disturbance	14.0 ± 5.9	12.7 ± 5.9	10.9 ± 5.2^†^	8.5 ± 3.7*	16.5 ± 4.1	12.3 ± 6.3	14.2 ± 6.8	15.2 ± 5.5
Physical symptoms	10.7 ± 5.3	13.1 ± 6.3	10.3 ± 4.8	9.3 ± 4.5	10.5 ± 5.7	13.8 ± 6.2	11.2 ± 5.6	14.8 ± 6.6
Emotional symptoms	9.0 ± 4.4	9.3 ± 4.7	7.4 ± 1.7	8.2 ± 4.7	8.5 ± 4.8	11.3 ± 5.3	10.4 ± 4.9	8.6 ± 4.1
Daytime function	9.1 ± 4.1	9.6 ± 4.6	9.4 ± 4.4	7.5 ± 3.5	9.5 ± 4.1	10.5 ± 4.4	8.7 ± 4.2	10.1 ± 5.0
Caregiver concerns	11.5 ± 6.1	13.3 ± 7.6	10.0 ± 4.8	8.8 ± 4.1^†^	12.5 ± 5.9	16.9 ± 7.8	11.6 ± 6.9	13.5 ± 7.9
Total score	54.5 ± 21.3	58.0 ± 23.0	47.9 ± 15.6	42.4 ± 14.1*^†^	57.4 ± 20.3	64.8 ± 22.1	56.6 ± 25.4	62.2 ± 24.4
*ABAS-II*								
		DS (*N* = 38)		DS (*N* = 11)		DS (*N* = 11)		DS (*N* = 16)
Practical composite		50.3 ± 13.9		49.8 ± 11.8		55.0 ± 19.2		47.5 ± 10.8
Social composite		69.8 ± 13.5		73.7 ± 13.9		69.3 ± 13.4		67.6 ± 13.6
Conceptual composite		55.7 ± 9.8		53.1 ± 7.3		58.6 ± 13.0		55.4 ± 8.7
General adaptive composite		52.8 ± 11.3		53.0 ± 9.6		55.6 ± 15.2		50.8 ± 9.4
*Pediatric Sleep Problem Survey Instrument*								
		DS (*N* = 38)		DS (*N* = 11)		DS (*N* = 10)		DS (*N* = 17)
Sleep routine		54.6 ± 10.6		52.3 ± 10.5		59.0 ± 10.6		53.5 ± 10.6
Bedtime anxiety		56.7 ± 12.2		53.0 ± 10.9		58.6 ± 10.6		57.9 ± 13.8
Morning tiredness		52.6 ± 10.8		49.7 ± 8.6		55.7 ± 14.9		52.5 ± 9.4
Night arousal		55.4 ± 13.2		51.1 ± 7.9		60.3 ± 16.6		55.2 ± 13.4
Restless sleep		58.7 ± 9.8		57.4 ± 9.9		60.3 ± 14.8		58.7 ± 5.9
Sleep-disordered breathing		69.9 ± 14.3		62.7 ± 9.0		66.3 ± 14.2		76.6 ± 14.4*
*Epworth Sleepiness Scale for Children and Adolescents*								
		DS (*N* = 33)		DS (*N* = 9)		DS (*N* = 8)		DS (*N* = 16)
Total score		5.4 ± 4.4		4.3 ± 3.4		4.5 ± 5.1		6.4 ± 4.6

Values are mean ± SD.

*p < 0.05 primary snoring vs moderate/severe OSA.

†*p* < 0.05 primary snoring vs mild OSA.

In the children with DS, there were no correlations between the number, duration, density or intensity of either spindle type and internalizing, externalizing or total problems of the CBCL. In contrast, in the TD children, spindle number, density and intensity for C Slow were positively correlated with internalizing problems (*p* < 0.05 for all) and total problems (*p* < 0.01 for all) and C Slow&Fast were also positively correlated with internalizing problems (*p* < 0.05 for all) and total problems (*p* < 0.01 for all). In addition, C Slow spindle density and intensity were correlated with externalizing problems (*p* < 0.05 for both) on the CBCL.

In the children with DS, spindle number, density and intensity for C Fast (*p* < 0.01 for all) and C Slow&Fast (*p* < 0.05 for all) were negatively correlated with OSA-18 emotional symptoms and caregiver concerns. C Fast number, density and intensity were also negatively correlated with daytime function (*p* < 0.01 for all) and total problems (*p* < 0.01 for all). There were no correlations identified for spindles recorded from frontal areas. In the TD children, the only correlation identified was for F Slow spindle density and intensity, which were positively associated with OSA-18 sleep disturbance (*p* < 0.05 for both).

Parents of children with DS also completed the ABAS-II, PSSI and ESS-CHAD. The practical composite score of the ABAS-II was negatively correlated with C Slow&Fast spindle intensity (*r* = −0.32, *p* < 0.05). On the PSSI, spindle density and intensity for C Fast (*r* = −4.1, *p* < 0.01) and C Slow&Fast (*r* = −0.34, *p* < 0.05) were negatively correlated with sleep routine and C Slow (*r* = −0.34, *p* < 0.05), C Fast (*r* = −0.34, *p* < 0.05) and C Slow&Fast (*r* = −0.35, *p* < 0.05) with night arousal indicating greater problems in these subscales were associated with fewer spindles. Total scores for the ESS-CHAD, indicating greater levels of sleepiness, were negatively correlated with C Slow and C Slow&Fast spindle number (*r* = −0.38, *p* < 0.05 for both), spindle density (*r* = −0.41 and *r* = −0.40 respectively, *p* < 0.05 for both) and spindle intensity (*r* = −0.40 and *r* = −0. 43, respectively, *p* < 0.05 for both).

## Discussion

Sleep spindles play an important role in protecting the sleeping brain from external sensory stimuli and can serve as markers of sleep integrity. This study identified that although children with DS spent more time in N2 sleep, where the majority of sleep spindles occur, they had significantly fewer and shorter Slow spindles recorded from frontal electrodes, with lower spindle density and intensity, compared to TD children. In addition, there were regional differences identified between children with DS and TD children. In TD children, there were more Slow compared to Fast spindles recorded on frontal electrodes with increased density and intensity in all SDB severity groups. In contrast, in the children with DS, the only differences identified in the density and intensity of Fast and Slow spindles were on central electrodes in those children with PS and MS OSA. Although there were no differences in CBCL or OSA-18 subscale scores between children with DS and TD children, we found different associations between spindle characteristics and the various subscales. In the children with DS, there were no correlations between the number, density or intensity of either spindle type with any of the CBCL subscales; in contrast, in the TD children, there were a number of positive correlations observed. Differences between groups of children were also present for correlations with the OSA-18 subscales, with children with DS exhibiting negative correlations and TD children positive. Our study has identified that sleep spindles are significantly reduced in children with DS and this reduction in sleep spindles may underpin the increased sleep disruption observed in these children and may underpin the negative effects of SDB on quality of life and behavior.

Despite spending more time in N2 sleep, where the majority of sleep spindles are found, the children with DS had fewer sleep spindles compared to matched TD children. When we separated children into SDB severity groups, the children with DS and Mild OSA had significantly fewer spindles than TD children with the same severity of SDB. In our previous study of a different cohort of TD children with and without SDB we also found that although spindle number, density and intensity were reduced in the children with SDB, this only reached statistical significance in the Mild OSA group.^31^ In that study, we had hypothesized that spindle numbers would be related to SDB severity; however, we found that spindle numbers were much more variable in the children with SDB and although there was a tendency for reduced spindles in the PS and MS OSA groups, this failed to reach statistical significance. Similarly, in this study, the variability in sleep spindle numbers, density and intensity was much greater in the children with DS and this likely affected our results. Although previous studies have not specifically examined sleep spindles in children with DS and SDB, an early study in adults with DS reported fewer sleep spindles.^37^ Studies in children with a spectrum of intellectual disabilities, including DS, have reported reduced sleep spindles compared to TD children, with a number of children having no sleep spindles and these children tended to have lower developmental quotients than children with sleep spindles.^38,39^ In our study two children with DS did not have any sleep spindles, whereas all TD children had spindles. Sleep spindles play a primary role in protecting the sleeping brain from external sensory stimuli and have been used as markers of sleep integrity.^10,11^ In addition, sleep spindles are thought to play an important role in the consolidation and re-organization of memories by preventing sleep fragmentation.^12,13^ The reduced number of sleep spindles in children with DS may explain the increased time spent awake after sleep onset we identified in this study, together with the increased prevalence of disorders of sleep maintenance reported in other studies.^18^ Sleep disruption has been suggested to predispose individuals with DS to earlier onset or faster deterioration of dementia.^40^ Alzheimer’s disease in adults has been associated with reduced spindle activity^17^ and impaired temporal coupling of slow wave activity and spindle activity.^41^ Given the almost universal incidence of Alzheimer’s disease in older adults with DS,^42^ our finding raises the possibility that sleep spindle activity could serve as a novel biomarker for early-onset Alzheimer’s disease in the DS population. Analysis of spindle activity in a larger group of children with DS would help to elucidate this connection. How the onset of dementia interacts with sleep disruption and the cognitive impacts of SDB is critically important information in informing the treatment of OSA in people with DS.

In general, there were few sleep macro-architecture differences between children with DS and TD children matched for SDB severity. However, we identified that in the group as a whole, and in those with MS OSA, children with DS spent more time in N2 sleep compared to their TD peers. There have been few PSG studies that have compared sleep microarchitecture in children with DS and matched TD children, and the findings differ between studies. In an early small study of 10 children with DS and various severities of SDB and 13 TD children with PS aged 1–10 years, although there was no difference in the percent time spent in N2 sleep the children with DS had shorter periods of N2.^43^ In addition, the children with DS had increased arousals from sleep and more sleep fragmentation, although it must be noted that the children with DS had more severe SDB.^43^ In a previous study by our group of a different cohort of children with DS aged 3–17 years (*N* = 32), there was no difference in time spent in N2 sleep, however time spent in N1 sleep was greater compared to TD children matched for SDB severity.^44^ In a larger group of children with DS (*N* = 130) aged 2–17 years, children aged 2–6 years (*N* = 30) spent less time in N2 and more time in N1 and N3, children 7–11 years (*N* = 36) spent more time in N3 and less time in REM and children aged 12–17 years (*N* = 17) spent less time in N1 and more time in N3 compared with age, sex and BMI matched TD children.^45^ Neither our previous study or the studies by Levanon et al. and Nisbet et al. reported WASO.^43–45^ In a study of 45 children with DS, time spent in N1 and REM was lower in children both under and over 6 years of age and time spent in N2 and N3 was greater in children over 6 years of age compared to that reported from normative data for age-matched children without SDB.^46^ Findings from the latter two studies suggest that alterations in sleep architecture are variable depending on the age of the children studied, but are likely to also be affected by SDB severity, which was not controlled for in the later study^46^ and which differed between age groups in the former study.^45^ In support of the findings of our current study, that children with DS spend more time awake after sleep onset, previous studies using parental questionnaire, actigraphy and pulse oximetry have also identified that children with DS had increased night awakenings and fragmented sleep together with lower peripheral oxygen saturation (SpO_2_), increased SpO_2_ dips and increased SpO_2_ variability compared to TD children;^47,48^ however, these studies could not quantify SDB severity. In a previous publication from this same group of children where we assessed sleep patterns in the home, we also identified that children with DS spent more time awake during the night than TD children.^31^ Although reduced sleep spindles may be contributing to more fragmented sleep in children with DS, our study did not find a relationship between spindle characteristics and SDB severity as assessed by either the OAHI or arousal index, suggesting that the finding of reduced spindles may be part of DS itself or due to a different cause.

A strength of our study was that we divided spindles into Fast and Slow and also assessed spindles from both the centroparietal and frontal EEG leads. Slow spindles predominate in frontal cortical areas, originate in the medial frontal region, and are associated with increased activation of the superior frontal gyrus. Fast spindles are dominant over central and parietal areas, originate in the precuneus, and are associated with the activation of the hippocampus, medial frontal cortex, and brain areas associated with sensory-motor processing.^8,49^ In both groups of children, there were more Slow compared to Fast spindles recorded from both frontal and centroparietal regions. In the TD children, there were more Slow compared to Fast spindles recorded on frontal electrodes with increased density and intensity in all SDB severity groups. In contrast, in the children with DS, the only differences identified in the density and intensity of Fast and Slow spindles were on central electrodes in those children with PS and MS OSA. The finding of fewer fast compared to slow spindles is supported by our previous study in a different cohort of TD children, with and without SDB,^31^ and previous studies in healthy non-snoring TD children.^14,16^ Frontal and centroparietal spindles exhibit different maturational patterns with the frequency of centroparietal spindles being relatively unchanged with age over childhood and adolescence, while frontal spindles become more prominent with age, with an increase in frequency around puberty.^50,51^ In the latter study, spindle density followed an inverted U-shape trajectory increasing to a peak at 12–14 years of age, before declining in late adolescence, with the authors suggesting that these changes were related to pubertal development.^51^ It has been suggested that increased spindle density may reflect increased myelination of thalamocortical projections,^52^ as neuroimaging has shown higher spindle density to be associated with enhanced white matter diffusion along axons.^53^ Frontal spindle activity in particular may be a good indicator of biological maturation of the central nervous system.^50^ Although there were differences in spindle density between our age-matched groups, the finding that both groups of children exhibited increased Slow spindles compared to Fast spindles and more frontal than centroparietal spindles suggests that the maturational patterns of sleep spindles are similar in children with DS. Sleep spindles have been associated with memory consolidation and brain plasticity in both TD children and children with neurodevelopmental disabilities.^54^ This raises the question as to whether stimulation of sleep spindle activity could improve sleep and cognition in children with DS. Transcranial direct current stimulation,^55,56^ intensive physical exercise,^57^ neurofeedback^58,59^ and some pharmacological manipulation of spindle density^60^ have been shown to increase spindle activity and improve sleep-dependent memory consolidation. It would be interesting to see if sleep spindle activity can be increased in children with DS and if this is associated with improved sleep and daytime functioning.

In their review, Gruber and Wise^13^ suggest that alterations in sleep spindles may interfere with cognition and behavior or alternatively some neurodevelopmental impairments and sleep spindle differences may both arise as independent manifestations of underlying brain abnormalities. We also identified differences in the associations between spindle characteristics and behavior and quality of life between children with DS and TD children. In the children with DS there were no correlations between spindle activity and measures on the CBCL. In contrast, in TD children, spindle number, density and intensity for C Slow and C Slow&Fast were positively correlated with internalizing problems and total problems and C Slow spindle density and intensity were correlated with externalizing problems on the CBCL. The finding in TD children seems counterintuitive, with more spindles being associated with worse behavior; we would have expected that the increased spindle activity would be related to better sleep and therefore better daytime behavior. Although previous studies in TD children have not examined the relationship between sleep spindles and behavior, they have reported differing results in regard to the direction and strength of associations between spindle characteristics and performance on intelligence and memory tasks. It could be that increased spindle activity represents a compensatory response, as has been suggested in Huntington’s disease.^61^ In the children with DS, where we found no relationship between spindle activity and behavior, other factors may be more important in determining daytime functioning, or alternatively the children with DS may not be able to mount any compensatory response due to altered brain development.

In the children with DS, there were a number of significant negative correlations between centroparietal Fast spindle activity and subscales of the OSA-18. This suggests that increased spindle activity is associated with improved daytime function, emotional symptoms, caregiver concerns and total problems. In contrast, in the TD children, the only correlation found was a positive relationship between spindle activity and sleep disturbance, suggesting as with the OSA-18 questionnaire, more spindles were related to more sleep disturbance. Previous studies have not compared spindle indices to the OSA-18, which is a specific quality-of-life questionnaire for children with SDB; thus, these findings need to be corroborated with future studies.

The parents of the children with DS also completed the PSSI, ABAS-II and ESS-CHAD, which assess sleep problems, the skills necessary for daily functioning, and daytime sleepiness, respectively. We identified that increased sleep problem scores were associated with reduced spindle activity. These findings suggest that the increased sleep disruption that we have previously reported compared to TD children^28^ may be mediated by reduced sleep spindles. The only correlation observed with the subscales of the ABAS-II was with C Slow&Fast intensity, which was negatively correlated with the Practical Composite Score. The majority of children scored in the extremely low range (scores <70) as expected, and as this correlation only just reached statistical significance (*r* = −0.32, *p* = 0.049), this may have been a chance finding underpinned by having only three subjects in the low range and 1 in the average range. Parents scored their children across a range of daytime sleepiness from 0 to 16, but consistently increased spindle activity was associated with lower levels of daytime sleepiness. This finding supports our contention that reduced sleep spindles are associated with more problematic sleep and increased sleepiness during the day.

In this study, sleep spindles were assessed manually following the protocol published by Chatburn et al.^14^ Our spindle numbers in TD children were lower than that reported by Chatburn et al.,^14^ who found a centroparietal spindle density of 0.3 Fast spindles/min and 2.3 Slow spindles/min in N2 sleep in children without SDB compared to our TD group 0.01 Fast spindles/min and 0.03–0.08 Slow spindles/min on from centroparietal electrodes during N2 and N3 sleep. This may have been because we also included spindles from N3 sleep, where there was a lower spindle density or it may have been because we were very rigorous in defining spindles and rejected those that were variable in amplitude and or frequency. All spindles included in the final analyses were assessed by a single researcher (MS) who was blinded to the group to avoid any selection bias. The numbers of spindles reported in children are very variable between studies and previous studies have reported fewer spindles in children with SDB compared to control children.^31,62,63^ To progress in this field of research, methods of standardization of spindle analysis need to be developed. Consistent across all studies is the finding that the numbers of spindles are variable among children even in control groups.^14,31,62–65^ In a large cross-sectional study, individual variation in spindle density was greatest in children 6–12 years compared to adolescents and young adults, reflecting maturational differences in thalamocortical networks and differences in pubertal development.^51^ This inter-subject variability likely reduced the power of our study to detect group differences. Whether spindle density has the potential to be a biomarker of future cognitive decline is an intriguing possibility.^41^

We acknowledge the limitations of our study. First, our sample size in each SDB severity group was small; however, this sample size has been previously reported as being adequately powered.^62^ Our TD and SDB groups were however well matched with no differences in demographics, SDB severity, sleep characteristics or questionnaire measures. Our spindle analysis was verified by a single researcher to minimize selection bias when identifying spindles. Second, the PSG was only performed on a single night and sleep may have been disrupted compared to sleep at home; however, both groups of children were studied in the same manner. Finally, we did not include a group of children with DS without SDB, so cannot separate the effects of SDB and DS itself on spindle density.

In summary, this is the first study to identify reduced sleep spindle activity in children with DS compared to TD children matched for SDB severity, age and sex. This finding may underpin the increased sleep problems and contribute to the cognitive differences identified in these children. Future studies should examine spindle density over a wider age range of children with DS and its relationship to neurocognitive and behavioral functioning. The potential for enhancement of sleep spindles to impact cognitive outcomes in these children holds promise for future therapeutic interventions.

### Supplementary Information

Supplementary information

## Data Availability

The datasets generated during and analyzed during the current study are available from the corresponding author upon reasonable request.
