# Supplementary material for: Sleep spindles are reduced in children with Down syndrome and sleep-disordered breathing
Source: Pediatr Res. 2023 Oct 16;96(2):457–70. doi: 10.1038/s41390-023-02854-1 (PMC11343711; doi:10.1038/s41390-023-02854-1)
Supplement: Supplementary file 1 — Supplementary information [file 41390_2023_2854_MOESM1_ESM.pdf]

**Online Supplementary Information.**

## Methods

### *Subjects*

Each child with Down syndrome (DS) was matched to a typically developing (TD) child for age and sex, and with the same SDB severity based on the obstructive apnea hypopnea index (OAHI): primary snoring (OAHI ≤1 event/hour), mild OSA (OAHI of >1-≤5 events/hour), moderate OSA (OAHI of >5-≤10 events/hour) or severe OSA (>10 events/hour). Children with DS with severe OSA were matched as closely as possible for OAHI. For children where a close match for all 3 parameters (OAHI, age and sex) was not possible, the match for OAHI was prioritized, then age, and the other sex was allowed (2 children in the Mild OSA group). If more than one TD child matched to a child with DS for all 3 parameters, the one with the closest OAHI was selected. The matching was performed prior to any analysis.

Parents completed a medical history form and demographics questionnaire. All TD children were born at term and were otherwise healthy with no comorbidities such as craniofacial syndromes, developmental disability or genetic syndromes as assessed by the referring pediatrician. Both TD and children with DS were well at the time of the PSG study and none of the TD children were taking medications known to affect sleep or breathing. Of the children with DS, two had undergone surgery for non-cyanotic congenital heart disease as an infant but were considered to have no active cardiac disease at the time of the study, four had undergone an adenotonsillectomy, six were on thyroxine for hypothyroidism and one was taking melatonin for sleep-onset insomnia.

### *Polysomnography*

The polysomnography (PSG) montage included electroencephalogram (EEG) (Cz, F3-M2, F4-M1, C3-M2, C4-M1, O1-M2, O2-M1), right and left electrooculogram (EOG), submental

electromyogram (EMG), left and right anterior tibialis muscle EMG, and electrocardiogram (ECG). Respiratory characteristics were captured using abdominal and thoracic respiratory plethysmography (Pro-Tech zRIP™ Effort Sensor, Pro-Tech Services Inc., Mukilteo, WA), oronasal thermistor, nasal pressure and transcutaneous carbon dioxide ($TcCO_2$), (TCM4/40, Radiometer, Denmark, Copenhagen or Sentec, Therwil, Switzerland). Peripheral oxygen saturation ($SpO_2$) was measured using Bitmos GmbH (Bitmos, Dusseldorf, Germany), which uses Masimo signal extraction technology for signal processing or Masimo Radical 7 (Masimo, Irving, CA), with both devices set to a 2-s averaging time.

***Sleep and respiratory analysis***

All PSG studies were scored manually in 30 s epochs for sleep stages (N1, N2, N3 and REM), respiratory events > 2 breaths in duration and arousals by trained pediatric sleep scientists using Compumedics ProFusion software according to American Academy of Sleep Medicine pediatric guidelines [1]. An obstructive apnea was defined as the cessation of airflow in association with ongoing respiratory effort; an obstructive hypopnea was defined as ≥30% decrease in nasal pressure signal amplitude, associated with increased work of breathing and an arousal or ≥3% decrease in oxygen saturation; a central apnea was defined as cessation of airflow without inspiratory effort lasting either ≥20 seconds or at least the duration of two breaths and associated with an arousal or ≥3% oxygen desaturation; and a central hypopnea as ≥30% decrease in nasal pressure signal amplitude with reduced inspiratory effort throughout the entire duration of the event. A mixed apnea was defined if an event was associated with absent respiratory effort during one portion of the event and the presence of obstructed inspiratory efforts in another portion, regardless of which portion came first [1]. The OAHI, defined as the total number of obstructive apneas, mixed apneas, and obstructive hypopneas per hour of total sleep time (TST) was used to define SDB severity. Other respiratory parameters included the respiratory disturbance index (RDI), defined as the total number of

respiratory events including obstructive and central apneas, mixed apneas, obstructive and central hypopneas; the REM RDI; the arousal index (ArI), defined as the number of cortical EEG arousals per hour of TST; the central apnea hypopnea index (CnAHI), defined as the number of central apneas and hypopneas per hour of TST. Desaturation measures included the average $SpO_2$ drop, defined as the average $SpO_2$ desaturation with scored respiratory events; $SpO_2$ nadir, the lowest oxygen saturation associated with a respiratory event; ODI 4%, defined as the number of times the $SpO_2$ dropped by greater than or equal to 4% per hour of TST; ODI 90%, defined as the number of times the $SpO_2$ dropped below 90% per hour of TST; and the average transcutaneous $pCO_2$ during TST (Av $TcCO_2$).

***Sleep Macro-architecture analysis***

Standard measures of sleep quality were calculated for each participant and included the following parameters. The duration of each sleep stage (N1, N2, N3, REM) was expressed as a % of TST. Wake after sleep onset (WASO) was calculated as the percentage of time awake during the sleep period time (SPT), defined as the amount of time in minutes from sleep onset until lights on at the end of the study, including all periods of wake in between. TST was defined as SPT excluding all periods of wake. Time in bed (TIB) was defined as the time between lights off and lights on. Sleep latency was defined as the period from lights off to the first 3 consecutive epochs of N1 sleep or an epoch of any other stage. Sleep efficiency was defined as the ratio of TST to TIB.

***Sleep spindles analysis***

Sleep spindles were identified manually using Compumedics Profusion 3.0 software (Melbourne, Australia). Spindles were identified from the C4-A1 and F4-A1 EEG channels during N2 and N3 sleep from raw EEG signals recorded using a band-pass filter of 0.3 Hz to 100 Hz and a sampling frequency of 512 Hz. Each 30 s epoch was studied with the EEG gain set to 25 $\mu$V per mm, the high pass band filtered at 0.3 Hz and low pass band filtered at 35 Hz

[2]. Spindles were categorized according to criteria reported by Chatburn et al, where identified spindles were required to be relatively equal in amplitude and with waves of a consistent frequency and a narrow conical shape with a duration of > 0.5 s [3]. Candidate spindles were not included if the amplitudes of the waves were not consistent and the waves were not conical, as if waves were too broad, the minimum of 5 peaks within 0.5 s would not be met and if too variable then this was most likely to be artefact. Waveforms that could otherwise be defined as a vertex or sawtooth wave or of highly variable amplitudes or frequencies were also excluded [3]. Spindles were then categorized and labelled as either being a F4 or C4 Slow or F4 or C4 Fast spindles, and F4 or C4 Slow&Fast spindles combined. Slow spindles consisted of 5-6 peaks while fast spindles had ≥7 peaks within 0.5 s. Broken spindles (spindles within 1 s of each other) were counted as one spindle [3].

Total spindle number was the total number of each category of spindle found in N2 and N3 sleep. The duration of every individual spindle was automatically recorded by Profusion and when exported, verified to have a duration >0.5 s before calculating the average duration for each spindle category. Spindle density was calculated as the number of spindles per minute of N2 or N3 sleep; spindle intensity was calculated as the product of spindle density and average spindle duration. The majority of spindles were identified in N2 as expected, but as including spindles from N3 increased the number of children with spindles in the various categories, data are presented with spindles from N2 and N3 combined.

***Questionnaires.***

The OSA-18 assesses quality of life in children with OSA [4]. It comprises 18 questions categorized into five domains: sleep disturbance, physical symptoms, emotional symptoms, daytime function and caregiver concerns. Scores are rated using a Likert scale about how frequently the child has experienced symptoms in the previous four weeks with scores ranging from “1 - None of the time” to “7 - All of the time.” Scores <60 suggest a small impact, scores

between 60 and 80 suggest a moderate impact and scores >80 suggest a significant impact on QOL [5].

The Child Behavior Checklist (CBCL) [6] for children 6 - 18 years old contains 118 questions. Scales include internalizing and externalizing behavior and total problems [7]. Questions are rated on a Likert scale as either 0 (not true), 1 (somewhat or sometimes true) or 2 (very true or often true). The raw scores were calculated by totaling the items under each scale and then converting to normal-referenced T-scores. T- scores ≥ 70, which are equivalent to scores above the 98th percentile, are considered to be of clinical concern [8].

In addition, the parents of the children with DS also completed the Adaptive Behavior Assessment System, Second Edition (ABAS II) [9], the Pediatric Sleep Problem Survey Instrument (PSSI) [10] and the Epworth Sleepiness Scale for Children and Adolescents (ESS-CHAD) [11].

The ABAS-II was used to assess skills necessary for daily functioning with separate versions used for children aged under 5 years of age and those over 5 years. The ABAS-II has three major adaptive domains: conceptual, social and practical tasks, covering eleven skill areas. Each skill area is scored according to a 4-point Likert scale with scores rated as 0 (is not able to), 1 (never when needed), 2 (sometimes when needed) or 3 (always when needed). The general adaptive composite is a composite score of all adaptive skill areas. Scaled scores for the three adaptive domains and general adaptive composite scores are classified as very superior (130 or more), superior (120-129), above average (110-119), average (90-109), below average (80-89), low (71-79) and extremely low (70 or less)[9].

The PSSI contains 26 questions assessing a range of sleep related disorders in children which are rated on a 4-point Likert scale of “Never,” “Rarely - once a week,” Sometimes – 2 to 4 times a week,” or “Usually – 5 to 7 times a week.” The relative items are summed and scores are assigned to assess subscales: sleep routine, bedtime anxiety, morning tiredness, night

arousals, sleep disordered breathing (SDB) and restless sleep. A T-score of ≥70, which is equivalent to a score above the 95th percentile, is indicative to be of clinical concern [10].

The ESS-CHAD is a measure of daytime sleepiness. It contains eight common daily situations, for which the parent scores how likely their child is to doze or fall asleep from a scale of 0 (would never doze or sleep) to 3 (high chance of dozing or sleeping). The total scores are summed and a score ≥ 10 suggests excessive daytime sleepiness [12].

**References**


1. Berry RB, *et al.* Rules for scoring respiratory events in sleep: Update of the 2007 aasm manual for the scoring of sleep and associated events. Deliberations of the sleep apnea definitions task force of the american academy of sleep medicine. J Clin Sleep Med 2012;8:597-619.
2. Shetty M, *et al.* The effects of sleep disordered breathing on sleep spindle activity in children and the relationship with sleep, behavior and neurocognition. Sleep Med 2023;101:468-77.
3. Chatburn A, *et al.* Sleep spindle activity and cognitive performance in healthy children. Sleep 2013;36:237-43.
4. Franco RA, Jr., Rosenfeld RM, Rao M. Quality of life for children with obstructive sleep apnea. Otolaryngol Head Neck Surg 2000;123:9-16.
5. Goldstein NA, Fatima M, Campbell TF, Rosenfeld RM. Child behavior and quality of life before and after tonsillectomy and adenoidectomy. Arch Otolaryngol Head Neck Surg 2002;128:770-5.
6. Achenbach TM 1999 The child behavior checklist and related instruments. . In Mahwah NJ (ed) The use of psychological testing for treatment planning and outcomes assessment. Lawrence Erlbaum Associates Publishers, USA, pp 429-66.
7. Eisenhower AS, Baker BL, Blacher J. Preschool children with intellectual disability: Syndrome specificity, behaviour problems, and maternal well-being. J Intellect Disabil Res 2005;49:657-71.
8. Marino M, *et al.* Distribution and age of onset of psychopathological risk in a cohort of children with down syndrome in developmental age. Ital J Pediatr 2019;45:92.

9. Oakland T, Harrison PL 2008 Adaptive behavior assessment system-ii.

10. Biggs SN, *et al.* Psychometric properties of an omnibus sleep problems questionnaire for school-aged children. Sleep Med 2012;13:390-5.

11. Janssen KC, *et al.* Validation of the epworth sleepiness scale for children and adolescents using rasch analysis. Sleep Medicine 2017;33:30-5.

12. Bergeron M, *et al.* The impact of persistent pediatric obstructive sleep apnea on the quality of life of patients' families. Int J Pediatr Otorhinolaryngol 2020;129:109723.
